# Two cases of unusual airway pathology, in which a careful history contributed to a successful diagnosis

**DOI:** 10.7196/AJTCCM.2018.v24i4.221

**Published:** 2018-12-20

**Authors:** S T Hlophe, R Masekela

**Affiliations:** Department of Paediatrics and Child Health, Nelson R Mandela School of Clinical Medicine, University of KwaZulu-Natal, Durban, South Africa

**Keywords:** wheezing, intensive care unit

## Abstract

Lower airway obstruction commonly presents with wheezing but is not always caused by asthma. Considering the case history and course of illness is of utmost importance in determining the cause of wheezing. We present two cases admitted to the paediatric intensive care unit, in which a double aortic arch was found to be the cause of wheezing. The cases illustrate the importance of a systematic approach when investigating a patient with persistent wheeze, especially when there is a poor response to conventional therapy.

## Background

Obstruction in the lower airways may occur in the trachea, bronchi or bronchioles.^[1]^ The most common symptom of lower airway obstruction is wheezing, caused by vibration of the airways during passage of air through a narrow lumen.^[1]^ Not all children who present with a bilateral wheeze have asthma. Considering the case history and the course of illness is of utmost importance in determining the cause of wheezing. Some anatomical, physiological and developmental factors make children particularly susceptible to airway obstruction.^[2,3]^ Respiratory distress, which accounts for 10% of visits to the paediatric emergency department, is more common in children than in adults because of children’s unique anatomical and physiological features.^[4]^ We present two unusual cases of wheezing admitted to the paediatric intensive care unit (PICU).

## Case 1

**Fig. 1:**
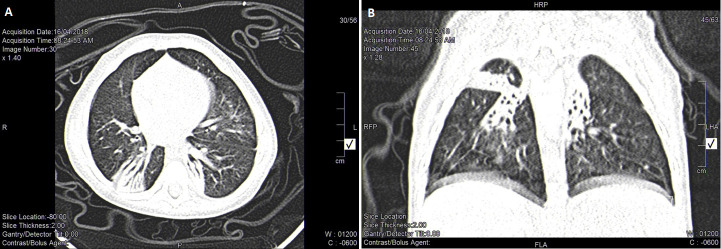
(A) Axial and (B) sagittal computed tomography images of the chest showing consolidation and subsegmental atelectasis in the right and left upper lobe, and consolidation and atelectasis, with tubular bronchiectasis, in the apical regions of the lower lobe.

A 10-month-old male patient was admitted to the PICU at the Inkosi Albert Luthuli Central Hospital (IALCH), Durban, with persistent upper and lower airway obstruction. He was born at 36 weeks following an uneventful pregnancy, with a birth weight of 2.49 kg. His history showed recurrent difficulty in breathing, vomiting and coughing after feeds since the age of 2 months. There was no family history of atopy and no history of choking episodes during meals. On anthropometric examination, he was found to be underweight for his age (Z-score <–3 SD). On examination, we found mild acute respiratory distress syndrome (partial oxygen pressure:fraction of inspired oxygen=205 on admission), stridor and wheeze. He was initiated on inhaled bronchodilator therapy and antireflux medication. As there was no evidence of gastro-oesophageal reflux disease following both a barium swallow and pH monitoring, the patient did not require any surgical intervention. An uncontrasted computed tomography (CT) scan was performed, which revealed bronchopneumonic changes with collapse and consolidation (Fig. 1).

**Fig. 2:**
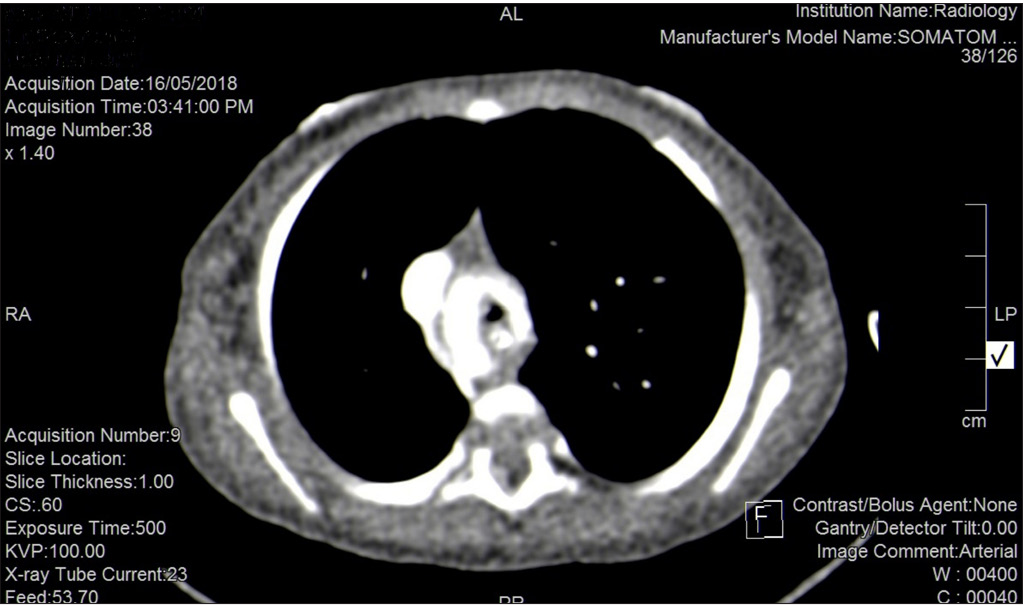
Axial computed tomography angiogram demonstrating an incomplete aortic sling noted as encasing both the trachea and oesophagus, with an impression of a fibrotic band or incomplete arterial communication in the quadrant between three o’clock and six o’clock.

A paediatric surgical team were consulted for an antireflux procedure (Nissen fundoplication). This was deferred owing to the patient’s clinical instability: he had refractory wheeze, carbon dioxide retention and was difficult to ventilate owing to lower airway obstruction despite the use of inhaled salbutamol and intravenous infusions of various agents (ketamine, aminophylline, salbutamol and magnesium sulphate). As the patient’s symptoms did not resolve, a chest CT scan with angiography was repeated. This revealed a double aortic arch (DAA) vascular ring, with separate origins on the left and right common carotid and subclavian arteries as seen from the respective arms of the aortic sling (Fig. 2). The anomaly was successfully repaired. Intraoperatively it was also noted that the patient had an oesophageal perforation, which was subsequently repaired. He was extubated successfully 6 days post operation.

## Case 2

**Fig. 3:**
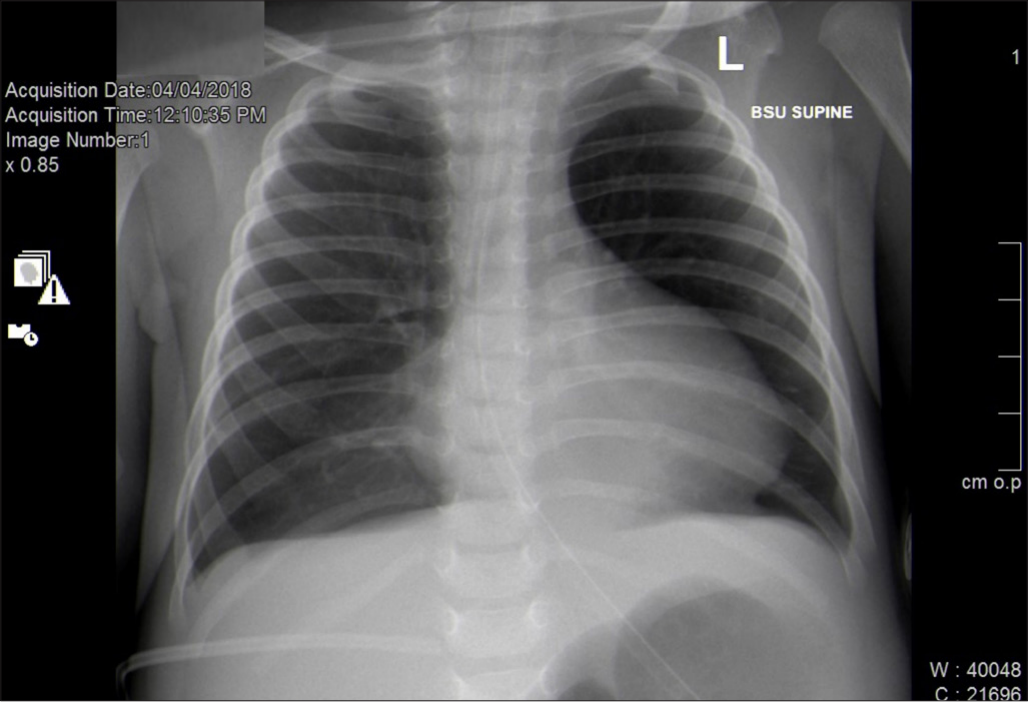
Chest X-ray, showing poor visualisation of the airway and hyperinflation.

A 3-month-old male patient presented to the PICU at IALCH with persistent upper and lower airway obstruction. He was born at full term after an uneventful pregnancy, with a birth weight of 3.2 kg. His medical history showed recurrent admissions for hoarseness of voice and blockage of the throat since birth. An IALCH otolaryngologist’s initial assessment via flexible laryngoscope at age 7 weeks revealed moderate laryngomalacia, which was treated conservatively. On admission to the PICU, the patient required ventilation owing to severe respiratory distress and upper airway obstruction. He failed extubation twice owing to obstruction in both the upper and lower airways. After the second failed extubation, examination of the airway under anaesthesia revealed glottic oedema, without laryngotracheomalacia. A tracheostomy was performed to relieve upper airway obstruction. The patient subsequently developed refractory lower airway obstruction, with no response to bronchodilators, and required prolonged infusions of aminophylline, ketamine, magnesium sulphate and rocuronium to relieve the bronchospasm. These episodes occurred sporadically, with sudden episodes of severe bronchospasm. Review of his chest X-rays revealed significant narrowing of the airways and a structural anomaly was suspected (Fig. 3).

**Fig. 4:**
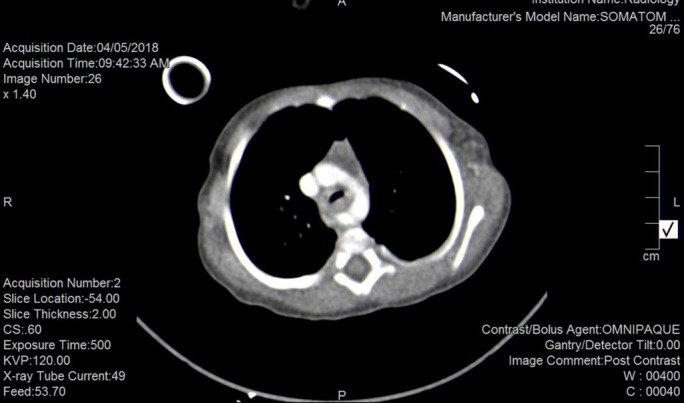
Axial computed tomography chest image showing a complete aortic sling, which appears to encase the trachea and oesophagus.

Chest CT revealed a DAA vascular ring encasing both the trachea and the oesophagus (Fig. 4). A division was performed successfully via left thoracotomy. The patient was extubated 6 days later and discharged.

## Discussion

Lower airway obstruction in children causes severe respiratory distress and may lead to respiratory failure and other complications of hypoxia. Different causes are listed in Table 1.

**Table 1:** Causes of large-airway obstruction in children

**Infection**
• Viral: respiratory syncytial virus bronchiolitis; parainfluenza; influenza; adenovirus; rhinovirus;
human metapneumovirus
• Bacterial: epiglottitis; tracheitis; tonsillitis; abscess adjacent to airway; pneumonia
• Congenital abnormalities: choanal atresia; choanal stenosis; micrognathia; macroglossia; laryngomalacia;
laryngeal web; vascular ring
**Cardiac disease**
• Congestive cardiac failure
• Myocarditis
• Cardiomyopathy
• Asthma
**Depressed level of consciousness**
**Gastro-oesophageal reflux**
**Foreign body**
**Trauma**
**Neoplasm: haemangioma; lymphoma; mediastinal mass**
**Peripheral neurological disease**
**Iatrogenic: subglottic stenosis; post-intubation stridor; neck haematoma**
**Anaphylactic reactions**

A thorough history and examination can contribute to a differential diagnosis. Diagnosis of a foreign body in the airway is based on positive history, a high index of suspicion and radiological signs such as the presence of a radio-opaque foreign body, unilateral emphysema, atelectasis and mediastinal shift. In a child with acute-onset wheezing, together with a history of preceding fever and abdominal pain and a temperature >38 °C at presentation, pneumonia is the likely diagnosis.^[1]^ Bilateral wheezing in children or infants is not necessarily due to bronchial asthma; asthma and congenital heart disease can coexist.^[1]^ In a young infant (2 - 6 months) with mild to moderate fever and wheeze associated with respiratory distress, bronchiolitis is the most likely diagnosis.^[1]^ Other causes of respiratory distress include the presence of a vascular ring,^[5]^ which was the cause of obstruction in both our cases.

### Double aortic arch

The occurrence of a DAA is a rare congenital vascular malformation due to the persistence of both the left and the right aortic arches after birth.^[6]^ The arches wrap the trachea and strangulate it, causing airway obstruction.^[6]^ Early diagnosis of a DAA is difficult owing to its rare occurrence.^[6]^ Children with a DAA usually present in infancy, with symptoms that include dysphagia, stridor, wheezing and respiratory distress.^[7]^ Tracheal compression may cause significant morbidity and mortality owing to severe hypoxia.^[8]^ Some cases present late, with tracheomalacia or lung complications.

Guidelines by the American Thoracic Society suggest that box-like flow-volume loops should alert clinicians to possible airway obstruction, with the reason for this functional respiratory compromise to be investigated through endoscopic and radiological approaches.^[9]^ A definitive diagnosis of DAA is usually achieved with radiological studies.^[8]^ A chest X-ray may show a deviation or compression of the trachea or the contour of a right arch.^[8]^ CT and magnetic resonance imaging provide a more accurate diagnosis.^[8]^ Surgical correction of the non-dominant arch is required to relieve the airway compromise.^[7]^ Division of the ring relieves compression of the trachea and oesophagus and the patient is weaned off the ventilator as soon as possible, as was evident in both our cases.

## Conclusion

The two cases described here demonstrate the challenges faced in managing airway obstruction. Both cases illustrate the importance of a systematic approach when investigating a patient with persistent wheeze, especially when there is a poor response to conventional therapy. Presence of a vascular ring should be suspected in a patient who presents with persistent lower airway obstruction, stridor and dysphagia when other aetiologies have been excluded. The first case also illustrates the importance of appropriate imaging, such as chest CT or CT angiography, for vascular abnormalities.
